# Increased Brain Age Among Psychiatrically Healthy Adults Exposed to Childhood Trauma

**DOI:** 10.1002/brb3.70450

**Published:** 2025-04-01

**Authors:** Chanellé Hendrikse, Leigh Luella van den Heuvel, Robin Emsley, Soraya Seedat, Stefan du Plessis

**Affiliations:** ^1^ Department of Psychiatry Stellenbosch University Cape Town South Africa; ^2^ Genomics of Brain Disorders Research Unit South African Medical Research Council/Stellenbosch University Cape Town South Africa

**Keywords:** adults, brain age, childhood trauma, machine learning, magnetic resonance imaging, sexual abuse

## Abstract

**Background:**

Adults with childhood trauma exposure may exhibit brain changes typically associated with aging and neurodegeneration (e.g., reduced tissue volume or integrity) to a greater degree than their unexposed counterparts, suggesting accelerated brain aging. Machine learning methods that predict a person's age based on their magnetic resonance imaging (MRI) brain scan may be useful for investigating aberrant brain aging following childhood trauma exposure. Emerging evidence indicates altered brain aging in adolescents with childhood trauma exposure; however, this association has not been examined in healthy adults.

**Methods:**

We investigated the associations between childhood trauma exposure, including abuse and neglect, and brain‐predicted age in psychiatrically healthy adults. “Brain age” predictions were generated from T1‐weighted structural MRI scans using a pre‐trained machine learning pipeline, namely brainageR. The differences between brain‐predicted age and chronological age were calculated and associations with childhood trauma questionnaire scores were investigated using linear regression.

**Results:**

The final sample (*n* = 153; mean age 46 ± 16 years, 70% female) included 69 adults with childhood trauma exposure and 84 unexposed adults. Childhood sexual abuse was associated with an average increased brain age of 3.2 years, adjusting for chronological age and age‐squared, sex, and scanner site; however, this finding did not survive correction for multiple comparisons.

**Conclusions:**

To our knowledge, this study represents the first published investigation of brain age in adults with childhood trauma using a machine‐learning‐based prediction model. Our findings suggest a link between childhood trauma exposure, specifically sexual abuse, and accelerated brain aging in adulthood, but this association should be replicated in future work. Accentuated brain aging in adulthood may increase the risk of age‐related cognitive and neurodegenerative decline and associated disorders later in life.

## Graphical abstract

We investigated the associations between childhood trauma exposure, including subtypes of abuse and neglect, and brain age in psychiatrically healthy adults. Brain age was predicted from T1‐weighted structural MRI scans using a machine‐learning‐based algorithm. Childhood sexual abuse was associated with increased brain age.

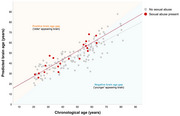

## 1 Introduction

Childhood trauma exposure, including experiences of abuse and neglect, is associated with an increased risk of psychopathology, including anxiety, depression, and substance abuse (Carr et al. 2013, Curran et al. 2018, Mclaughlin et al. 2012, McLaughlin and Lambert 2017, McCrory et al. 2017). It has also been linked with poorer cognitive performance (Petkus et al. 2018) and neurodegenerative disorders later in life (Corney et al. 2022, Xie et al. 2023, Subramanian et al. 2023). This elevated risk may be underpinned by stress‐induced HPA‐axis dysregulation and brain changes which endure throughout life (Teicher and Samson 2016a, Teicher and Samson 2016b). Excessive exposure to stress hormones during childhood may disrupt crucial processes underlying healthy neurodevelopment, such as synaptic pruning, myelination, and neurogenesis (Gunnar and Quevedo 2007). Growing evidence suggests that the deleterious impact of stress on brain development may also have downstream negative effects on neurophysiological processes linked with brain aging, including telomere erosion, impaired DNA repair, mitochondrial dysfunction, oxidative stress, and neuroinflammation (Chaudhari et al. 2022, Mattson and Arumugam 2018). These neurodevelopmental and physiological aging aberrations may influence trajectories of brain tissue (i.e., grey and white matter) maturation and degeneration across the lifespan, and manifest as brain structural or functional changes in individuals with childhood trauma.

Substantive literature has demonstrated an association between childhood trauma exposure and altered structure, connectivity, and function of stress‐susceptible brain regions or systems (McCrory et al. 2017, Teicher and Samson 2016b, Teicher and Samson 2016a, McLaughlin et al. 2014). Neuroimaging studies have reported smaller global and regional grey and white matter volumes (Begemann et al. 2023, Teicher et al. 2012), greater cortical thinning (McLaughlin et al. 2014), and reduced fractional anisotropy in specific white matter tracts in individuals with childhood trauma (Hendrikse et al. 2024, Cunha et al. 2021, Tendolkar et al. 2018, Lim et al. 2020). These brain changes are consistent with commonly described age‐related neurodegenerative brain changes in older adults (Bethlehem et al. 2022), and may be indicative of accelerated brain aging in individuals exposed to childhood trauma. Depending on the severity and rate of deterioration, accelerated brain aging may lead to increased morbidity and premature mortality (Cole et al. 2018, Cole and Franke 2017). Studies have reported an increased prevalence or earlier onset of neurodegenerative disorders such as Alzheimer's disease (Corney et al. 2022), Parkinson's disease (Subramanian et al. 2023), and dementia (Xie et al. 2023) among individuals with a history of childhood trauma.

Magnetic resonance imaging (MRI) can be used in a variety of ways to study brain structure. Common methods include voxel‐based morphometry, cortical parcellation or subcortical segmentation, and white matter tractography. These methods have provided valuable insights into the potential impact of childhood trauma on specific structural properties of whole brain or predefined regions of interest. In recent years, machine learning techniques have emerged that can be used to estimate a person's age based on their MRI brain scan (Cole et al. 2017, Cole et al. 2019). These methods analyze brain structural features in a cumulative manner across the brain. By comparing these features against age‐labelled datasets, the algorithms learn to predict age with a high degree of accuracy. Notably, a person's brain‐predicted age may differ substantially from his or her chronological age. This discrepancy is often termed the ‘brain‐predicted age difference’ or ‘brain‐PAD’. Brain age predictions show promise as a potential biomarker for accelerated aging and risk of neurodegenerative diseases (Soumya Kumari and Sundarrajan 2024).

Few studies have used machine‐learning‐based brain age prediction methods to investigate aberrant brain aging in individuals exposed to early‐life adversity, including childhood trauma. The majority of these studies have been in child/adolescent samples with mixed findings depending on the type of early adversity or trauma studied. For example, a cumulative measure of early‐life environmental adversity has been linked to ‘older’ brain age (i.e., positive brain age gap) in adolescents (Drobinin et al. 2022). Another study found an association between childhood abuse and a ‘younger’ appearing brain, specifically in emotion circuitry, in contrast to brain‐wide increased brain age seen with physical neglect (Keding et al. 2021). A recent longitudinal multimodal MRI study reported younger‐looking brains in adolescents exposed to emotional neglect and older‐looking brains in adolescents exposed to other adverse exposures, including caregiver psychopathology and family aggression (Beck et al. 2025). Despite discrepant findings, these studies provide evidence of an association between childhood trauma and altered patterns of brain maturation during adolescence. With the exception of a single unpublished study which found an association between certain sensitive periods of exposure to childhood trauma and increased brain age in adult women (Fleming et al. 2024), the influence of childhood trauma on brain aging beyond adolescence, as determined with machine learning methods, has not been examined.

Using a validated brain age prediction model (Cole et al. 2017), we investigated brain age in adults with childhood trauma exposure. Considering that distinct forms of childhood trauma, such as abuse and neglect, may have differential effects on neurodevelopment (McLaughlin et al. 2014) and brain aging, as demonstrated in the adolescent literature (Beck et al. 2025), we also examined brain age associations with different dimensions (e.g., abuse and neglect) and subtypes (e.g., physical and sexual abuse) of childhood trauma. Our sample included psychiatrically healthy adults only in comparison to prior studies that have examined brain aging in patient samples with psychiatric disorders (Jha et al. 2023, Clausen et al. 2022). We hypothesized that overall childhood trauma exposure, or specific trauma dimensions or subtypes, would be associated with increased brain age (Fleming et al. 2024).

## 2 Materials and Methods

### 2.1 Study Design and Participants

**FIGURE 1:**
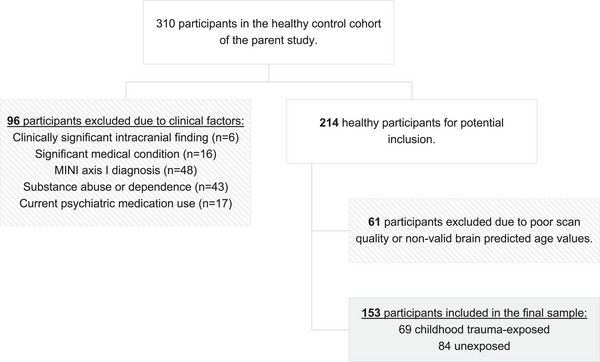
Flowchart of participant exclusions.

Participants were drawn from the healthy control group (*n* = 310) of a cross‐sectional study (Grant Number: MRC‐RFA‐IFSP‐01‐2013/SHARED ROOTS). Approval for the study was obtained from the Health Research Ethics Committee of the Faculty of Medicine and Health Sciences at Stellenbosch University (Ethics Approval Number: HREC N13/08/115). Participation in the study was voluntary, and all participants provided written informed consent. Clinical assessments and brain imaging were performed between 2014 and 2017. Figure 1 presents a flowchart of participant exclusions which resulted in a final sample of 153 adults (72% female, aged between 20 and 81 years).

### 2.2 Clinical Assessments

Participants were screened for major medical (e.g., HIV, cancer) and psychiatric disorders and concomitant medications by completing a general medical questionnaire, recording previous and concomitant medications, and a diagnostic interview with the MINI International Neuropsychiatric Interview, version 6.0 (Sheehan et al. 1997). Metabolic syndrome status was determined based on the harmonized joint interim statement (JIS) criteria described elsewhere (Alberti et al. 2009). Briefly, three of the following risk factors are required for a positive diagnosis: hypertension, elevated triglycerides, abnormal cholesterol, abnormal fasting glucose or diabetes, and elevated waist circumference. Metabolic syndrome status was compared between groups since obesity (Ronan et al. 2016) and certain lifestyle factors, such as less physical exercise (Steffener et al. 2016), have been linked with increased brain age; moreover, being overweight has been linked with brain structural changes (Cole et al. 2013).

### 2.3 Childhood Trauma Assessment

Childhood trauma was assessed with the 28‐item version of the Childhood Trauma Questionnaire (CTQ; Bernstein et al. 2003). The CTQ is a widely used and reliable self‐report scale measuring early‐life exposure to five trauma subtypes including physical, emotional, and sexual abuse and physical and emotional neglect (Bernstein et al. 2003). A CTQ total scale score (ranging from 25 to 125) and five subscale scores (ranging from 5 to 25) were calculated for each participant. Composite scale scores were also derived for overall abuse (sum of the abuse‐specific subscales, ranging from 15 to 75) and overall neglect (sum of the neglect‐specific subscales, ranging from 10 to 50).

The total CTQ scale, composite, and subscale scores were used in the main statistical analyses for brain age. However, for descriptive and interpretive purposes, participants were additionally categorized by the level of exposure to each childhood trauma subtype according to established subscale‐specific thresholds (Bernstein and Fink 1998, Bernstein et al. 2003). The presence of childhood trauma exposure overall was defined as a score above the “moderate” threshold on any one of five CTQ subscales.

Internal consistency of the CTQ and subscales was generally good in this sample (Cronbach's alpha: overall trauma = 0.904, emotional abuse = 0.838, physical abuse = 0.840, sexual abuse = 0.916, emotional neglect = 0.871). The physical neglect subscale had the lowest Cronbach's alpha of 0.460, consistent with previous studies in other populations (He et al. 2019, Petrikova et al. 2021). It is possible that the interpretation of some of the items on this scale (e.g., ‘I did not have enough to eat’, ‘I had to wear dirty clothes’) were confounded by experiences of extreme poverty. Nevertheless, pooling the emotional and physical neglect subscale items, as has been previously done (Peng et al. 2023), resulted in an acceptable Cronbach's alpha of 0.860. Therefore, these subscales were combined into a broader ‘overall neglect’ category in subsequent analyses.

### 2.4 Assessment of Lifetime Exposure to Stressful Events

Apart from childhood trauma, exposure to additional stressful or traumatic events in childhood or later life (i.e., cumulative lifetime traumas) may augment lifetime psychopathology risk and influence brain outcomes (da Silva et al. 2024). Therefore, some past studies examining the associations between childhood trauma exposure and brain outcomes have excluded participants with multiple unrelated forms of adversity, including natural disasters, motor vehicle accidents, house fires, near drownings, combat or war exposure, gang violence or murder, assault with a weapon, etc. (Andersen et al. 2008, Teicher et al. 2012, Teicher et al. 2018). Cumulative lifetime trauma exposure was not an exclusionary factor in the present study, as this would have resulted in a much smaller sample size. Therefore, the Life Events Checklist for DSM‐5 (LEC‐5; 48) was used to assess lifetime history of potentially traumatic events, for example, natural disasters, serious accidents, physical or sexual assault, combat or war‐zone exposure, or sudden, unexpected loss of a loved one. The average number of types of LEC‐5‐events experienced was compared between CT‐exposed and ‐unexposed participants. To examine the impact of the cumulative lifetime traumas on the associations between childhood trauma and brain‐PAD, post hoc sensitivity analyses were conducted which additionally adjusted for the total number of types of LEC‐5‐events experienced.

### 2.5 Neuroimaging Data Acquisition

High‐resolution T1‐weighted MEMPRAGE brain scans were acquired on one of two research‐dedicated MRI scanners: a 3T Siemens Allegra situated at the Cape Universities Brain Imaging Centre (CUBIC) Tygerberg, or a 3T Siemens Skyra situated at the CUBIC, University of Cape Town. Ninety‐one participants were scanned on the Allegra scanner with sequence parameters: TR = 2530 ms, TE_1_ = 1.53 ms, TE_2_ = 3.21 ms, TE_3_ = 4.89 ms, TE_4_ = 6.57 ms, flip angle = 7 degrees, FoV = 256 mm, 128 slices, 1 mm isotropic voxel size. Sixty‐two participants were scanned on the Skyra scanner with sequence parameters: TR = 2530 ms, TE_1_ = 1.63 ms, TE_2_ = 3.47 ms, TE_3_ = 5.31 ms, TE_4_ = 7.15 ms, flip angle = 7 degrees, FoV = 280 mm, 128 slices, 1 mm isotropic voxel size. All scans were screened for intracranial pathology by a radiologist and neurologist. Participants with clinically significant intracranial pathology were appropriately referred for further medical examination or treatment and excluded from this analysis.

### 2.6 Brain Age Prediction

All scans were visually inspected for sufficient quality before generating brain‐predicted age values. Quality exclusions were performed in a blinded manner and did not result in a childhood trauma exposure difference between participants with usable scans versus participants with unusable scans. We used a pre‐trained machine learning pipeline, brainageR version 2.1, to generate brain‐predicted age values from participants’ raw T1‐weighted MRI scans (Cole et al. 2017). The brainageR model was trained on 3377 healthy adults between the ages of 18 and 92 years and is freely accessible online (https://github.com/james‐cole/brainageR). BrainageR uses a Gaussian processes regression and is implemented in R. It invokes SPM12 for initial segmentation of cortical and subcortical grey matter, white matter, and cerebrospinal fluid, and normalization steps. The pipeline steps have been described previously (Biondo et al. 2022). Notably, brainageR applies a voxel‐wise method to parcellate grey matter, white matter, and CSF. This approach has been shown to predict brain age with comparable, or in some cases improved (Clausen et al. 2022) accuracy compared with other methods (Bacas et al. 2023).

### 2.7 Model Validation

As the brain age prediction model was not trained on our own data, we assessed the performance of the model in our sample. We calculated the mean absolute error (MAE) and Pearson correlation coefficient between predicted brain age and chronological age, along with the proportion of the variance explained by the model for the final sample (*n* = 153) (Han et al. 2021).

### 2.8 Statistical Analysis

Statistical analysis was performed in SPSS. Descriptive statistics were run to characterize the sample socio‐demographics and assess potential group differences between childhood trauma‐exposed versus‐unexposed participants (two‐tailed independent‐samples *t* tests for continuous variables and two‐sided Pearson chi‐square tests for categorical variables). Thereafter, the total, composite, and CTQ subscale scores were used in linear regression models to investigate the associations of overall childhood trauma and distinct trauma types with brain aging. The dependent variable in all models was brain‐predicted age difference (brain‐PAD), calculated as the difference between the predicted brain age and chronological age for each participant. A negative brain‐PAD value represents a ‘younger’ appearing brain, whereas a positive brain‐PAD value represents an ‘older’ appearing brain. Covariates in all models were chronological age, age‐squared, sex, and scanner site. Chronological age was included as a covariate to correct for residual age effects on the brain‐PAD variable, whereas quadratic age was included as the model tends to overestimate brain‐PAD at older ages and underestimate brain‐PAD at younger ages in a non‐linear manner (Le et al. 2018). Sex was included as a covariate considering accumulating evidence showing a differential impact of childhood trauma on stress‐related biology and brain structure in males and females (Tiwari and Gonzalez 2018). Scanner site was included as is the custom in multisite neuroimaging studies (Clausen et al. 2022, Han et al. 2021). The false discovery rate (FDR) method was applied to correct for multiple comparisons.

## 3 Results

### 3.1 Sample Socio‐Demographics

The final sample comprised 69 healthy adults with a history of childhood trauma and 84 without a history of childhood trauma. These groups were balanced in terms of age, sex, educational level, income, scanner site, and metabolic syndrome status (Table 1).

**TABLE 1:** Sample characteristics: Childhood trauma‐exposed versus unexposed participants.

	Final sample (*N* = 153)		
Variable^a^	CT‐exposed group (*n* = 69)	CT‐unexposed group (*n* = 84)	Statistics
Age, years	45 ± 16 (21–81)	48 ± 16 (20–80)	*t* (151) = 1.213, *p* = 0.227
Sex, female	51 (74)	56 (67)	*X^2^* (1) = 0.946, *p* = 0.331
Educational level Primary school (nearly) completed Partial secondary school Secondary school completed Any tertiary education	5 (7) 42 (61) 14 (20) 8 (12)	5 (6) 39 (46) 31 (37) 9 (11)	*X^2^* (3) = 5.171, *p =* 0.160
Monthly income^b^ < ZAR 3000 ZAR 3000–ZAR 6000 > ZAR 6000	29 (43) 18 (27) 20 (30)	32 (40) 21 (26) 27 (34)	*X^2^* (2) = 0.273, *p =* 0.872
Scanner site			*X^2^* (1) = 2.781, *p =* 0.095
3T Siemens Allegra 3T Siemens Skyra	36 (52) 33 (48)	55 (65) 29 (35)	
Metabolic syndrome status	17	22	*X^2^* (1) = 0.048, *p =* 0.826

Abbreviations: CT, childhood trauma; ZAR, South African rand.

^a^ Values for continuous variables are presented as: mean ± standard deviation (range). Values for categorical variables are presented as: number (%).

^b^ Missing income data for *n* = 2 CT‐exposed and *n* = 4 CT‐unexposed participants.

### 3.2 Overlap Between Childhood Trauma Subtypes

Table 2 presents information about the composite and distinct trauma categories for the childhood trauma group (*n* = 69). Additionally, it presents the *p*‐values for bivariate correlations between subscale scores for the CTQ. As expected, exposure to different trauma subtypes were highly correlated with each other.

**TABLE 2:** Overlap between childhood trauma subtypes and sub‐sample characteristics.

	*n* in final sample	% in CT‐E group	% female	Age, years	Brain age, years	Correlation with additional trauma types (p‐values)			
Trauma type						Emotional abuse	Physical abuse	Sexual abuse	Overall neglect
*Composite trauma categories*									
Any childhood trauma	69	100	74	45 ± 16 (21–81)	45 ± 13 (24–78)	—	—	—	—
Any abuse	60	87	75	43 ± 15 (21–79)	43 ±13 (24–78)	—	—	—	—
Any neglect	29	42	76	49 ± 16 (23–81)	48 ± 14 (24–77)	—	—	—	—
*Distinct trauma types*									
Emotional abuse	49	71	78	41 ± 15 (21–80)	42 ± 12 (24–78)	—	< 0.001	< 0.001	< 0.001
Physical abuse	27	39	67	42 ± 14 (23–78)	43 ± 12 (24–67)	< 0.001	—	0.005	< 0.001
Sexual abuse	20	29	90	43 ± 15 (21–63)	46 ± 13 (26–68)	< 0.001	0.005	—	< 0.001
Overall neglect	29	42	76	49 ± 16 (23–81)	48 ± 14 (24–77)	< 0.001	< 0.001	< 0.001	—

Abbreviation: CT‐E, childhood trauma‐exposed.

### 3.3 Lifetime Exposure to Stressful Events

As expected, the CT‐exposed group reported significantly higher rates of lifetime physical or sexual assault exposure (both *p* < 0.001), as well as a significantly higher average number of types of LEC‐5‐events experienced (CT‐exposed group: *M* = 6.03; CT‐unexposed group: *M* = 4.04; *p* < 0.001).

### 3.4 Brain Age Prediction Model Performance

The brain age prediction model predicted chronological age with a MAE of 6.34 ± 5.07 years in the final sample (*n* = 153), which is consistent with other studies (Han et al. 2021). Moreover, predicted brain age and chronological age were highly correlated in the final sample (*r* = 0.860, *p* < 0.001, *R^2^ =* 0.74), demonstrating adequate model performance.

### 3.5 Childhood Trauma Exposure and Brain Age

Overall childhood trauma (i.e., CTQ total score), overall abuse, and overall neglect were not associated with altered brain‐PAD (childhood trauma: β = 0.023, SE = 0.042, *p* = 0.592; abuse: β = 0.091, SE = 0.069, *p* = 0.190; neglect: β = 0.074, SE = 0.088, *p* = 0.403). In a post‐hoc linear regression model, we examined the unique associations of distinct childhood trauma subtypes (i.e., physical abuse, sexual abuse, emotional abuse, and overall neglect) with brain‐PAD, similarly adjusting for age, age‐squared, sex, and scanner site. The CTQ subscale scores for these trauma types were entered simultaneously in the model as done previously to determine the specific effect of each individual trauma type (Hendrikse et al. 2024, Hendrikse et al. 2022). Childhood sexual abuse had a statistically significant positive effect on brain‐PAD (β = 0.325, SE = 0.139, *p* = 0.021, 95% CI 0.050, 0.601); however, this finding did not survive FDR correction for multiple comparisons (adjusted *p* = 0.084). Sensitivity analysis additionally adjusting for the number of types of LEC‐5‐events experienced did not alter the significance of this finding (β = 0.358, SE = 0.140, *p* = 0.011, 95% CI 0.082, 0.635). The full results are reported in the Supporting Information.

**FIGURE 2:**
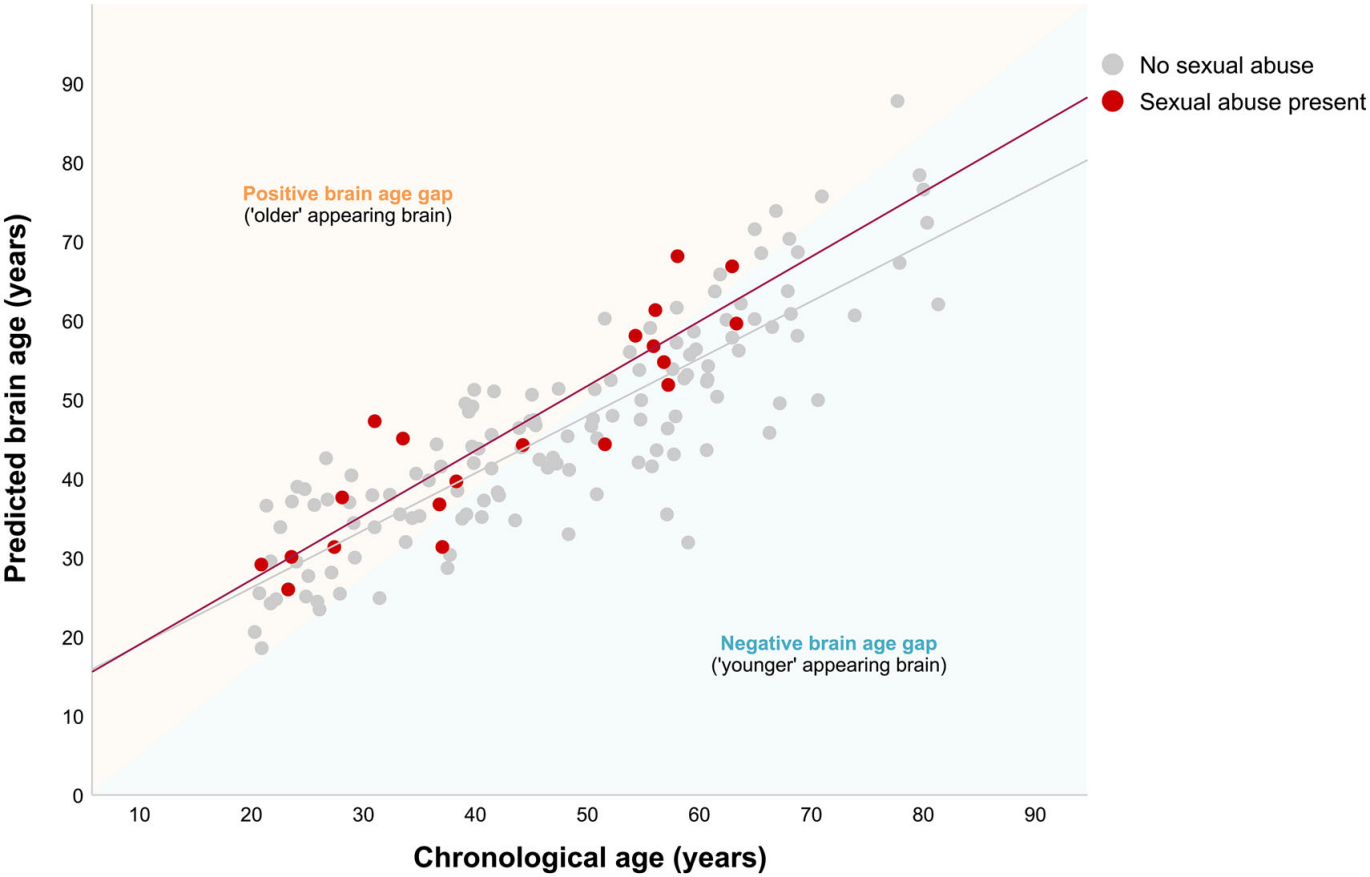
Scatterplot of brain age predictions for participants with sexual abuse (red dots) and without sexual abuse (grey dots). The diagonal line where the orange and blue triangles meet represents perfect prediction accuracy. Dots above the line (orange triangle) represent predicted brain age older than chronological age. Dots below the line (blue triangle) represent predicted brain age younger than chronological age. Seventy‐five percent of participants with sexual abuse had a positive brain age gap (i.e., an ‘older’ appearing brain).

Notably, 15 out of 20 (i.e., 75%) participants with a history of child sexual abuse—compared with 65 out of 133 (49%) participants without a history of child sexual abuse—exhibited positive brain‐PAD values, suggesting accentuated brain aging (Figure 2). Moreover, the average brain age of participants with a history of childhood sexual abuse was 3.2 years greater than their average chronological age. The overall model explained a significant portion of the variance in brain‐PAD (R‐square = 0.34, i.e., 34%, *p *< 0.001). Except for age and age squared which exhibited high collinearity in all models, the assumption of linearity was not violated.

## 4 Discussion

This study represents the first published investigation of brain age in adults with childhood trauma using a machine‐learning‐based prediction model. The applied model was found to predict age with a high level of accuracy in our sample, lending support to the validity of our findings. We found an association between childhood sexual abuse and a positive brain‐PAD in psychiatrically healthy adults, indicating increased brain age of approximately 3 years. While this association did not survive correction for multiple comparisons, it is consistent with the previous reports of altered brain aging in adolescents with childhood trauma or adversity (Drobinin et al. 2025, 2022. 2025, Keding 2021), as well as the evidence that distinct forms of childhood trauma differentially impact brain aging (Beck et al. 2025, Keding 2021). Moreover, recent meta‐analytic evidence also points to an association between accelerated biological aging in terms of cellular aging and earlier pubertal timing in children with a history of abuse specifically (Colich et al. 2020).

However, meaningful interpretation of the current results is challenging due to a lack of peer‐reviewed studies on the effects of childhood trauma on brain age in psychiatrically healthy adults. Nevertheless, the findings from animal studies may provide some insight. The animal literature points to key physiological processes that may influence the trajectory of brain aging, including mitochondrial and oxidative stress, proteostatic and epigenetic changes, and neuroinflammation (Chaudhari et al. 2022). It should be noted, however, that the preponderance of literature in this area has been correlational, with very few lifespan or longitudinal studies (Chaudhari et al. 2022). To our knowledge at the time of writing this paper, no other published study has used machine learning to predict brain age in adult participants with childhood trauma. However, one unpublished study, reported as conference proceedings, provided evidence that certain sensitive periods during adolescence were associated with an  impact of childhood trauma on accelerated brain aging in adult women (Fleming et al. 2024). Specifically, parental physical and verbal abuse and witnessing sibling abuse during pre‐ and/or early‐adolescence were associated with increased brain age. Their findings highlight an important limitation of the present study, namely that we did not investigate the impact of timing of exposure to childhood trauma on brain age. Past studies concerning brain morphological changes following childhood trauma have also reported sensitive periods for augmented impact of exposure (Teicher et al. 2018).

Incidentally, we observed that all participants with a history of childhood sexual abuse in this study were under the age of 65 years. In typically‐aging individuals, white matter volume decreases and cerebrospinal fluid volume increases begin around the age of 40 years and significantly accelerate after the age of 50 (Bethlehem et al. 2022, van Blooijs et al. 2023). Therefore, our finding suggests that the potential accelerative effects of childhood sexual abuse on brain aging may become evident relatively soon after tissue‐degenerative processes begin or accelerate. This may be because childhood trauma (i.e., excessive stress hormone exposure) impacts neurophysiological processes underlying patterns of brain maturation or aging soon after exposure, as the adolescent literature suggests. Therefore, our finding of older brain age among individuals with childhood sexual abuse may represent a long‐standing pathological pattern of brain aging. Even though our findings preliminarily suggest a potential enduring impact of childhood trauma on brain aging in adulthood, further replication and longitudinal work are needed to investigate trajectories of delayed or accelerated brain maturation and aging across the lifespan following childhood trauma, as well as how aberrant brain aging may be associated with increased risk of neurodegenerative diseases. Moreover, future work should elucidate how commonly reported structural and functional brain changes in individuals with childhood trauma (e.g., grey matter volumetric and functional changes in cortico‐striatal‐limbic regions and white matter integrity changes in fronto‐limbic tracts; 10–11, 16–22) may contribute to patterns of aberrant brain aging.

Moreover, the relatively young age of participants with a history of childhood sexual abuse in our sample implies that there is more time for severe degeneration and subsequent cognitive decline and related comorbidities in older age. It is therefore also plausible that the accentuated brain aging observed in individuals with childhood sexual abuse in this sample might represent a putative clinical biomarker for pathological brain aging and the emergence or earlier onset of neurodegenerative disorders later in life (Clausen et al. 2022). This is supported by previous reports of increased prevalence of neurodegenerative disorders among individuals exposed to childhood trauma (Corney et al. 2022, Xie et al. 2023), including childhood sexual abuse (Widom et al. 2023).

Current brain age prediction methods are not without limitations. Notably, brain age predictions based on MRI scans may be suboptimal or unreliable due to MRI‐related artifacts in training and/or testing data (Soumya Kumari and Sundarrajan 2024, Jha et al. 2023). Additionally, in the present study, the use of a single imaging modality is another limitation (Clausen et al. 2022), as brain age predictions may be more accurate when multimodal imaging data are used (Cole 2020). For example, imaging phenotypes or metrics could be derived from multimodal MRI (e.g., T1‐weighted MRI, diffusion‐MRI, task fMRI) and used to determine the relative informative value of different phenotypes for predicting brain age, as in Cole (2020). Cole (2020) found that, in 2205 healthy people with multimodal neuroimaging data, multiple neuroimaging measures were informative for brain age prediction, however most measures related to grey matter volume and white matter microstructure. Moreover, when single modalities were investigated separately, T1‐weighted MRI data were the most accurate for predicting brain age. Nevertheless, because the methodology for predicting brain age from MRI scans is relatively new, there is currently limited understanding or direct knowledge of the exact or most predictive brain morphological features that inform the predictions (Soumya Kumari and Sundarrajan 2024), that is, the “black‐box” problem (Hassija et al. 2024). Indeed, brain age predictions may be somewhat non‐specific and represent a single composite metric of many features from regions across the entire brain and may differ between each person (Franke and Gaser 2019). It is therefore not currently possible to definitively determine the predictive value (if any) of potential brain structural changes that have been reported previously in individuals with a history of childhood trauma, for example, grey matter volumetric changes of stress‐sensitive cortical and subcortical regions and/or white matter integrity changes of fronto‐limbic tracts (Teicher and Samson 2016b, Teicher and Samson 2016a, Begemann et al. 2023, Teicher et al. 2012, McLaughlin et al. 2014, Hendrikse et al. 2024, Cunha et al. 2021, Tendolkar et al. 2018, Lim et al. 2020). While we acknowledge the importance of future work to explore the most predictive brain features of brain age estimations, this was beyond the scope of the present study. Nevertheless, brain age prediction may be useful in illustrating the negative effects of childhood trauma on overall brain health and as a biomarker for disease risk in clinical settings (Clausen et al. 2022).

A notable limitation of this study was the small sizes of the groups when the participants were stratified by exposure to trauma subtypes. While we found a significant effect for childhood sexual abuse exposure on brain‐PAD, it should be noted that only 20 participants reported a history of childhood sexual abuse in our sample, and this finding did not survive FDR correction for multiple comparisons. The small group sizes may mean that statistical power was limited in the current study. Nevertheless, considering the critical lack of studies that have used machine learning methods to study brain aging in adults with a history of childhood trauma, this study is vital to illustrate the importance of this topic and inspire future work with larger total and subgroup sample sizes.

A strength of this study was the inclusion of psychiatrically healthy participants. In contrast to prior studies that have linked a range of psychiatric conditions with accelerated brain aging (Clausen et al. 2022, Han et al. 2021, Constantinides et al. 2024), the intentional exclusion of individuals with serious clinical or psychiatric conditions in this study allowed us to investigate the link between childhood trauma exposure and accelerated brain aging, unconfounded by psychiatric illness. Our findings therefore demonstrate aberrant brain aging even in apparently resilient individuals with a history of childhood trauma, specifically sexual abuse, which may have clinical implications for healthy brain aging in older age. Moreover, our findings represent a potential biological basis for studies reporting accelerated cognitive decline in individuals with childhood trauma exposure. However, it should be noted that since our findings do not survive multiple comparisons correction, further replications are critical. Moreover, future studies should investigate the clinical and functional correlates of brain age in individuals with childhood trauma, as well as examine other metrics of accelerated biological aging in conjunction with machine learning‐based brain age estimations. Understanding the impact of childhood trauma on brain aging and health outcomes is essential for identifying vulnerable populations and implementing early interventions to promote healthy brain maturation and to mitigate adverse outcomes later in life.

## Author Contributions

**Chanellé Hendrikse**: conceptualization, methodology, formal analysis, investigation, data curation, visualization, writing–original draft. **Leigh van den Heuvel**: project administration, investigation, data curation, writing–review and editing. **Robin Emsley**: conceptualization, methodology, supervision, writing–review and editing. **Soraya Seedat**: conceptualization, methodology, funding acquisition, supervision, writing–review and editing. **Stefan du Plessis**: conceptualization, methodology, investigation, software, supervision, writing–review and editing.

## Ethics Statement

Approval for the study was obtained from the Health Research Ethics Committee of the Faculty of Medicine and Health Sciences at Stellenbosch University (Ethics Approval Number: HREC N13/08/115). Participation in the study was voluntary, and all participants provided written informed consent.

### Peer Review

The peer review history for this article is available at https://publons.com/publon/10.1002/brb3.70450

## Supporting information



Supporting Information

## Data Availability

Study data is available from the corresponding author upon reasonable request.

## References

[brb370450-bib-0001] Alberti, K. , R. H. Eckel , S. M. Grundy , et al. 2009. “Harmonizing the Metabolic Syndrome: A Joint Interim Statement of the international diabetes federation task force on epidemiology and prevention; National heart, lung, and Blood Institute; American heart association; World heart federation; International.” Circulation 120: 1640–1645. 19805654 10.1161/CIRCULATIONAHA.109.192644

[brb370450-bib-0002] Andersen, S. L. , A. Tomada , E. S. Vincow , E. Valente , A. Polcari , and M. H. Teicher . 2008. “Preliminary Evidence for Sensitive Periods in the Effect of Childhood Sexual Abuse on Regional Brain Development.” Journal of Neuropsychiatry and Clinical Neurosciences 20: 292–301. 18806232 10.1176/appi.neuropsych.20.3.292 PMC4270804

[brb370450-bib-0003] Bacas, E. , I. Kahhalé , P. R. Raamana , J. B. Pablo , A. S. Anand , and J. L. Hanson . 2023. “Probing Multiple Algorithms to Calculate Brain Age: Examining Reliability, Relations With Demographics, and Predictive Power.” Human Brain Mapping 44: 3481–3492. 37017242 10.1002/hbm.26292 PMC10203791

[brb370450-bib-0004] Beck, D. , L. Whitmore , N. Macsweeney , et al. 2025. “Dimensions of Early Life Adversity Are Differentially Associated With Patterns of Delayed and Accelerated Brain Maturation.” Biological Psychiatry 97, no. 1: 64–72. 39084501 10.1016/j.biopsych.2024.07.019

[brb370450-bib-0005] Begemann, M. J. H. , M. J. L. Schutte , E. Van Dellen , et al. 2023. “Childhood Trauma Is Associated With Reduced Frontal Gray Matter Volume: A Large Transdiagnostic Structural MRI Study.” Psychological Medicine 53: 741–749. 34078485 10.1017/S0033291721002087 PMC9975993

[brb370450-bib-0006] Bernstein, D. P. , and L. Fink . 1998. Childhood Trauma Questionnaire: A Retrospective Self‐Report Questionnaire and Manual. The Psychological Corporation.

[brb370450-bib-0007] Bernstein, D. P. , J. A. Stein , M. D. Newcomb , et al. 2003. “Development and Validation of a Brief Screening Version of the Childhood Trauma Questionnaire.” Child Abuse & Neglect 27: 169–190. 12615092 10.1016/s0145-2134(02)00541-0

[brb370450-bib-0008] Bethlehem, R. A. I. , J. Seidlitz , S. R. White , et al. 2022. “Brain Charts for the Human Lifespan.” Nature 604: 525–533. 35388223 10.1038/s41586-022-04554-y PMC9021021

[brb370450-bib-0009] Biondo, F. , A. Jewell , M. Pritchard , et al. 2022. “Brain‐age Is Associated With Progression to Dementia in Memory Clinic Patients.” NeuroImage: Clinical 36: 103175. 36087560 10.1016/j.nicl.2022.103175 PMC9467894

[brb370450-bib-0010] Carr, C. P. , C. M. S. Martins , A. M. Stingel , V. B. Lemgruber , and M. F. Juruena . 2013. “The Role of Early Life Stress in Adult Psychiatric Disorders: A Systematic Review According to Childhood Trauma Subtypes.” Journal of Nervous and Mental Disease 201, no. 12: 1007–1020. 24284634 10.1097/NMD.0000000000000049

[brb370450-bib-0011] Chaudhari, P. R. , A. Singla , and V. A. Vaidya . 2022. “Early Adversity and Accelerated Brain Aging: A Mini‐Review.” Frontiers in Molecular Neuroscience 15: 822917. 10.3389/fnmol.2022.822917. 35392273 PMC8980717

[brb370450-bib-0012] Clausen, A. N. , K. A. Fercho , M. Monsour , et al. 2022. “Assessment of Brain Age in Posttraumatic Stress Disorder: Findings From the ENIGMA PTSD and Brain Age Working Groups.” Brain and Behavior 12, no. 1: e2413. 10.1002/brb3.2413. 34907666 PMC8785613

[brb370450-bib-0013] Cole, J. H. 2020. “Multimodality Neuroimaging Brain‐age in UK Biobank: Relationship to Biomedical, Lifestyle, and Cognitive Factors.” Neurobiology of Aging 92: 34–42. 32380363 10.1016/j.neurobiolaging.2020.03.014 PMC7280786

[brb370450-bib-0014] Cole, J. H. , C. P. Boyle , A. Simmons , et al. 2013. “Body Mass Index, but Not FTO Genotype or Major Depressive Disorder, Influences Brain Structure.” Neuroscience 252: 109–117. 23933215 10.1016/j.neuroscience.2013.07.015

[brb370450-bib-0015] Cole, J. H. , and K. Franke . 2017. “Predicting Age Using Neuroimaging: Innovative Brain Ageing Biomarkers.” Trends in Neurosciences 40: 681–690. 29074032 10.1016/j.tins.2017.10.001

[brb370450-bib-0016] Cole, J. H. , R. E. Marioni , S. E. Harris , and I. J. Deary . 2019. “Brain Age and Other Bodily ‘Ages’: Implications for Neuropsychiatry.” Molecular Psychiatry 24: 266–281. 29892055 10.1038/s41380-018-0098-1 PMC6344374

[brb370450-bib-0017] Cole, J. H. , R. P. K. Poudel , D. Tsagkrasoulis , et al. 2017. “Predicting Brain Age With Deep Learning From Raw Imaging Data Results in a Reliable and Heritable Biomarker.” Neuroimage 163: 115–124. 28765056 10.1016/j.neuroimage.2017.07.059

[brb370450-bib-0018] Cole, J. H. , S. J. Ritchie , M. E. Bastin , et al. 2018. “Brain Age Predicts Mortality.” Molecular Psychiatry 23: 1385–1392. 28439103 10.1038/mp.2017.62 PMC5984097

[brb370450-bib-0019] Colich, N. L. , M. L. Rosen , E. S. Williams , and K. A. McLaughlin . 2020. “Biological Aging in Childhood and Adolescence following Experiences of Threat and Deprivation: A Systematic Review and Meta‐Analysis.” Psychological Bulletin 146, no. 9: 721–764. 10.1037/bul0000270.supp. 32744840 PMC7484378

[brb370450-bib-0020] Constantinides, C. , V. Baltramonaityte , D. Caramaschi , et al. 2024. “Assessing the Association Between Global Structural Brain Age and Polygenic Risk for Schizophrenia in Early Adulthood: A Recall‐by‐Genotype Study.” Cortex; A Journal Devoted to the Study of the Nervous System and Behavior 172: 1–13. 38154374 10.1016/j.cortex.2023.11.015

[brb370450-bib-0021] Corney, K. B. , E. C. West , S. E. Quirk , et al. 2022. “The Relationship between Adverse Childhood Experiences and Alzheimer's Disease: A Systematic Review.” Frontiers in Aging Neuroscience 14: 831378. 10.3389/fnagi.2022.831378. 35601624 PMC9115103

[brb370450-bib-0022] Cunha, P. J. , F. L. S. Duran , P. A. de Oliveira , et al. 2021. “Callosal Abnormalities, Altered Cortisol Levels, and Neurocognitive Deficits Associated With Early Maltreatment Among Adolescents: A Voxel‐Based Diffusion‐Tensor Imaging Study.” Brain Behavior 11: 1–9. 10.1002/brb3.2009 PMC7994704 33452751

[brb370450-bib-0023] Curran, E. , G. Adamson , M. Rosato , P. De Cock , and G. Leavey . 2018. “Profiles of Childhood Trauma and Psychopathology: US National Epidemiologic Survey.” Social Psychiatry and Psychiatric Epidemiology 53, no. 11: 1207–1219. 29725700 10.1007/s00127-018-1525-y

[brb370450-bib-0024] da Silva, H. C. , L. Vilete , E. S. F. Coutinho , et al. 2024. “The Role of Childhood Cumulative Trauma in the Risk of Lifetime PTSD: An Epidemiological Study.” Psychiatry Research 336: 115887. 10.1016/j.psychres.2024.115887. 38642421

[brb370450-bib-0025] Drobinin, V. , H. Van Gestel , C. A. Helmick , M. H. Schmidt , C. V. Bowen , and R. Uher . 2022. “The Developmental Brain Age Is Associated with Adversity, Depression, and Functional Outcomes among Adolescents.” Biological Psychiatry: Cognitive Neuroscience and Neuroimaging 7: 406–414. 34555562 10.1016/j.bpsc.2021.09.004

[brb370450-bib-0026] Fleming, L. , K. Ohashi , S. Katrinli , et al. 2024. “364. Timing of Exposure to Childhood Maltreatment Differentially Impacts Brain Age Acceleration Later in Life: Evidence for Sensitive Periods.” Biological Psychiatry 95: S248.

[brb370450-bib-0027] Franke, K. , and C. Gaser . 2019. “Ten Years of Brainage as a Neuroimaging Biomarker of Brain Aging: What Insights Have We Gained?” Frontiers in Neurology 10: 789. 10.3389/fneur.2019.00789. 31474922 PMC6702897

[brb370450-bib-0028] Gunnar, M. , and K. Quevedo . 2007. “The Neurobiology of Stress and Development.” Annual Review of Psychology 58: 145–173. 10.1146/annurev.psych.58.110405.085605 16903808

[brb370450-bib-0029] Han, L. K. M. , R. Dinga , T. Hahn , et al. 2021. “Brain Aging in Major Depressive Disorder: Results From the ENIGMA Major Depressive Disorder Working Group.” Molecular Psychiatry 26: 5124–5139. 32424236 10.1038/s41380-020-0754-0 PMC8589647

[brb370450-bib-0030] Hassija, V. , V. Chamola , A. Mahapatra , et al. 2024. “Interpreting Black‐Box Models: A Review on Explainable Artificial Intelligence.” Cognitive Computation 16: 45–74.

[brb370450-bib-0031] He, J. , X. Zhong , Y. Gao , G. Xiong , and S. Yao . 2019. “Psychometric Properties of the Chinese Version of the Childhood Trauma Questionnaire‐Short Form (CTQ‐SF) Among Undergraduates and Depressive Patients.” Child Abuse & Neglect 91: 102–108. 30856597 10.1016/j.chiabu.2019.03.009

[brb370450-bib-0032] Hendrikse, C. J. , S. du Plessis , H. K. Luckhoff , et al. 2022. “Childhood Trauma Exposure and Reward Processing in Healthy Adults: A Functional Neuroimaging Study.” Journal of Neuroscience Research 100: 1452–1462. 35434795 10.1002/jnr.25051 PMC9546243

[brb370450-bib-0033] Hendrikse, C. , H. K. Lückhoff , J. P. Fouché , et al. 2024. “Fronto‐Limbic White Matter Microstructural Changes in Psychiatrically Healthy Adults With Childhood Trauma.” Journal of Neuroscience Research 102, no. 2: e25308. 10.1002/jnr.25308. 38361421

[brb370450-bib-0034] Jha, M. K. , C. Chin Fatt , A. Minhajuddin , T. L. Mayes , and M. H. Trivedi . 2023. “Accelerated Brain Aging in Adults with Major Depressive Disorder Predicts Poorer Outcome with Sertraline: Findings from the EMBARC Study.” Biological Psychiatry: Cognitive Neuroscience and Neuroimaging 8: 462–470. 36179972 10.1016/j.bpsc.2022.09.006 PMC10177666

[brb370450-bib-0035] Keding, T. J. 2021. “Early‐Life Exposure to Violence and Fronto‐Amygdala Circuit Maturation: Developmental Markers of Psychiatric Risk.” PhD diss., The University of Wisconsin‐Madison.

[brb370450-bib-0036] Keding, T. J. , S. A. Heyn , J. D. Russell , et al. 2021. “Differential Patterns of Delayed Emotion Circuit Maturation in Abused Girls With and Without Internalizing Psychopathology.” American Journal of Psychiatry 178: 1026–1036. 34407623 10.1176/appi.ajp.2021.20081192 PMC8570983

[brb370450-bib-0037] Le, T. T. , R. T. Kuplicki , B. A. McKinney , et al. 2018. “A Nonlinear Simulation Framework Supports Adjusting for Age When Analyzing BrainAGE.” Frontiers in Aging Neuroscience 10: 1–11. 30405393 10.3389/fnagi.2018.00317 PMC6208001

[brb370450-bib-0038] Lim, L. , H. Howells , J. Radua , and K. Rubia . 2020. “Aberrant Structural Connectivity in Childhood Maltreatment: A Meta‐Analysis.” Neuroscience and Biobehavioral Reviews 116: 406–414. 32659288 10.1016/j.neubiorev.2020.07.004

[brb370450-bib-0039] Mattson, M. P. , and T. V. Arumugam . 2018. “Hallmarks of Brain Aging: Adaptive and Pathological Modification by Metabolic States.” Cell Metabolism 27, no. 6: 1176–1199. 29874566 10.1016/j.cmet.2018.05.011 PMC6039826

[brb370450-bib-0040] McCrory, E. J. , M. I. Gerin , and E. Viding . 2017. “Annual Research Review: Childhood Maltreatment, Latent Vulnerability and the Shift to Preventative Psychiatry—the Contribution of Functional Brain Imaging.” Journal of Child Psychology and Psychiatry and Allied Disciplines 58, no. 4: 338–357. 28295339 10.1111/jcpp.12713 PMC6849838

[brb370450-bib-0041] Mclaughlin, K. A. , G. G. Jennifer; , M. J. Gruber , N. A. Sampson , A. M. Zaslavsky , and R. C. Kessler . 2012. “Childhood Adversities and First Onset of Psychiatric Disorders in a National Sample of US Adolescents.” Archives of General Psychiatry 69, no. 11: 1151–1160. 23117636 10.1001/archgenpsychiatry.2011.2277 PMC3490224

[brb370450-bib-0042] McLaughlin, K. A. , and H. K. Lambert . 2017. “Child Trauma Exposure and Psychopathology: Mechanisms of Risk and Resilience.” Current Opinion in Psychology 14: 29–34. 27868085 10.1016/j.copsyc.2016.10.004 PMC5111863

[brb370450-bib-0043] McLaughlin, K. A. , M. A. Sheridan , and H. K. Lambert . 2014. “Childhood Adversity and Neural Development: Deprivation and Threat as Distinct Dimensions of Early Experience.” Neuroscience and Biobehavioral Reviews 47: 578–591. 25454359 10.1016/j.neubiorev.2014.10.012 PMC4308474

[brb370450-bib-0044] McLaughlin, K. A. , M. A. Sheridan , W. Winter , N. A. Fox , C. H. Zeanah , and C. A. Nelson . 2014. “Widespread Reductions in Cortical Thickness Following Severe Early‐Life Deprivation: A Neurodevelopmental Pathway to Attention‐deficit/Hyperactivity Disorder.” Biological Psychiatry 76: 629–638. 24090797 10.1016/j.biopsych.2013.08.016 PMC3969891

[brb370450-bib-0045] Olson, E. A. , T. A. Overbey , C. G. Ostrand , D. A. Pizzagalli , S. L. Rauch , and I. M. Rosso . 2020. “Childhood Maltreatment Experiences Are Associated With Altered Diffusion in Occipito‐temporal White Matter Pathways.” Brain and Behavior 10: e01485. 10.1002/brb3.1485. 31773917 PMC6955831

[brb370450-bib-0046] Peng, C. , J. Cheng , F. Rong , Y. Wang , and Y. Yu . 2023. “Psychometric Properties and Normative Data of the Childhood Trauma Questionnaire‐short Form in Chinese Adolescents.” Frontiers in Psychology 14: 1–9. 10.3389/fpsyg.2023.1130683 PMC10008899 36923147

[brb370450-bib-0047] Petkus, A. J. , E. J. Lenze , M. A. Butters , E. W. Twamley , and J. L. Wetherell . 2018. “Childhood Trauma is Associated With Poorer Cognitive Performance in Older Adults.” Journal of Clinical Psychiatry 79, no. 1: 16m11021. 10.4088/JCP.16m11021. PMC6959209 29228518

[brb370450-bib-0048] Petrikova, M. , N. Kascakova , J. Furstova , J. Hasto , and P. Tavel . 2021. “Validation and Adaptation of the slovak Version of the Childhood Trauma Questionnaire (Ctq).” International Journal of Environmental Research and Public Health 18: 1–12. 10.3390/ijerph18052440 PMC7967575 33801428

[brb370450-bib-0049] Ronan, L. , A. F. Alexander‐Bloch , K. Wagstyl , et al. 2016. “Obesity Associated With Increased Brain Age From Midlife.” Neurobiology of Aging 47: 63–70. 27562529 10.1016/j.neurobiolaging.2016.07.010 PMC5082766

[brb370450-bib-0050] Sheehan, D. V. , Y. Lecrubier , K. H. Sheehan , et al. 1997. “The Validity of the Mini International Neuropsychiatric Interview (MINI) According to the SCID‐P and Its Reliability.” European Psychiatry 12: 232–241.

[brb370450-bib-0051] Soumya Kumari, L. K. , and R. Sundarrajan . 2024. “A Review on Brain Age Prediction Models.” Brain Research 1823: 148668. 10.1016/j.brainres.2023.148668. 37951563

[brb370450-bib-0052] Steffener, J. , C. Habeck , D. O'Shea , Q. Razlighi , L. Bherer , and Y. Stern . 2016. “Differences Between Chronological and Brain Age Are Related to Education and Self‐reported Physical Activity.” Neurobiology of Aging 40: 138–144. 26973113 10.1016/j.neurobiolaging.2016.01.014 PMC4792330

[brb370450-bib-0053] Subramanian, I. , B. McDaniels , J. Farahnik , and L. K. Mischley . 2023. “Childhood Trauma and Parkinson Disease.” Neurology Clinical Practice 13, no. 2: e200124. 10.1212/cpj.0000000000200124. 36891464 PMC9987208

[brb370450-bib-0054] Teicher, M. H. , C. M. Anderson , K. Ohashi , et al. 2018. “Differential Effects of Childhood Neglect and Abuse During Sensitive Exposure Periods on Male and Female Hippocampus.” Neuroimage 169: 443–452. 29288867 10.1016/j.neuroimage.2017.12.055 PMC5856615

[brb370450-bib-0055] Teicher, M. H. , and J. A. Samson . 2016a. “Annual Research Review: Enduring Neurobiological Effects of Childhood Abuse and Neglect.” Journal of Child Psychology and Psychiatry and Allied Disciplines 57, no. 3: 241–266. 26831814 10.1111/jcpp.12507 PMC4760853

[brb370450-bib-0056] Teicher, M. H. , and J. Samson . 2016b. “The Effects of Childhood Maltreatment on Brain Structure, Function and Connectivity.” Nature Reviews Neuroscience 17: 652–666. 27640984 10.1038/nrn.2016.111

[brb370450-bib-0057] Teicher, M. , C. M. Anderson , and A. Polcari . 2012. “Childhood Maltreatment Is Associated With Reduced Volume in the Hippocampal Subfields CA3, Dentate Gyrus, and Subiculum.” Proceedings of the National Academy of Sciences 109: E563–E572. 10.1073/pnas.1115396109 PMC3295326 22331913

[brb370450-bib-0058] Tendolkar, I. , J. Mårtensson , S. Kühn , F. Klumpers , and G. Fernández . 2018. “Physical Neglect During Childhood Alters White Matter Connectivity in Healthy Young Males.” Human Brain Mapping 39: 1283–1290. 29250891 10.1002/hbm.23916 PMC6866381

[brb370450-bib-0059] Tiwari, A. , and A. Gonzalez . 2018. “Biological Alterations Affecting Risk of Adult Psychopathology Following Childhood Trauma: A Review of Sex Differences.” Clinical Psychology Review 66: 69–79. 29433843 10.1016/j.cpr.2018.01.006

[brb370450-bib-0060] van Blooijs, D. , M. A. van den Boom , J. F. van der Aar , et al. 2023. “Developmental Trajectory of Transmission Speed in the Human Brain.” Nature Neuroscience 26: 537–541. 36894655 10.1038/s41593-023-01272-0 PMC10076215

[brb370450-bib-0061] Weathers, F. W. , D. D. Blake , P. P. Schnurr , D. G. Kaloupek , B. P. Marx , and T. M. Keane . 2013. The Life Events Checklist for DSM‐5 (LEC‐5). National Center for PTSD.

[brb370450-bib-0062] Widom, C. S. , H. H. Do , K. S. Lynch , and J. J. Manly . 2023. “Childhood Maltreatment and Dementia Risk Factors in Midlife: A Prospective Investigation.” Current Alzheimer Research 20: 636–647. 38155463 10.2174/0115672050281539231222071355

[brb370450-bib-0063] Xie, Z. , M. Li , H. Sun , et al. 2023. “Childhood, Adulthood, and Cumulative Traumatic Events Experienced From Childhood to Adulthood and Dementia Risk: A Population‐based Cohort Study.” *Journal of Public Health*. 10.1007/s10389-023-02140-8.

