# Supplementary material for: Increased Brain Age Among Psychiatrically Healthy Adults Exposed to Childhood Trauma
Source: Brain Behav. 2025 Apr 1;15(4):e70450. doi: 10.1002/brb3.70450 (PMC11962057; doi:10.1002/brb3.70450)
Supplement: Supplementary file 1 — Supporting Information [file BRB3-15-e70450-s001.docx]

| **Supplementary Material: Full results of main models** | | | | | |
| --- | --- | --- | --- | --- | --- |
| **Dependent variable in all models: brain-predicated age difference (brain-PAD)** | | | | | |
|  | | | | | |
| **MODEL 1** | | | | | |
|  | **Unstandardized B** | **Coefficients Std. Error** | **Standardized Coefficients Beta** | **t** | ***p*** |
| *Trauma variable(s):* |  |  |  |  |  |
| CTQ total scale score | 0.023 | 0.042 | 0.038 | 0.537 | 0.592 |
| *Covariates:* |  |  |  |  |  |
| Age | -0.540 | 0.206 | -1.056 | -2.620 | 0.010 |
| Age squared | 0.003 | 0.002 | 0.484 | 1.189 | 0.236 |
| Sex | -1.838 | 1.265 | -0.104 | -1.453 | 0.148 |
| Scanner site | 2.255 | 1.158 | 0.137 | 1.946 | 0.054 |
|  | | | | | |
|  | | | | | |
| **MODEL 2** | | | | | |
|  | **Unstandardized B** | **Coefficients Std. Error** | **Standardized Coefficients Beta** | **t** | ***p*** |
| *Trauma variable(s):* |  |  |  |  |  |
| Abuse total score | 0.091 | 0.069 | 0.102 | 1.317 | 0.190 |
| Neglect total score | -0.074 | 0.088 | -0.064 | -0.838 | 0.403 |
| *Covariates:* |  |  |  |  |  |
| Age | -0.555 | 0.206 | -1.086 | -2.695 | 0.008 |
| Age squared | 0.003 | 0.002 | 0.527 | 1.293 | 0.198 |
| Sex | -1.652 | 1.272 | -0.094 | -1.299 | 0.196 |
| Scanner site | 2.082 | 1.164 | 0.126 | 1.788 | 0.076 |
|  | | | | | |
|  | | | | | |
| **MODEL 3** | | | | | |
|  | **Unstandardized B** | **Coefficients Std. Error** | **Standardized Coefficients Beta** | **t** | ***p*** |
| *Trauma variable(s):* |  |  |  |  |  |
| Physical abuse total | 0.033 | 0.164 | 0.017 | 0.203 | 0.839 |
| Sexual abuse total | 0.325 | 0.139 | 0.169 | 2.332 | 0.021 |
| Emotional abuse total | -0.084 | 0.191 | -0.037 | -0.441 | 0.660 |
| Overall neglect total | -0.076 | 0.088 | -0.066 | -0.870 | 0.386 |
| *Covariates:* |  |  |  |  |  |
| Age | -0.560 | 0.206 | -1.095 | -2.716 | 0.007 |
| Age squared | 0.003 | 0.002 | 0.522 | 1.280 | 0.203 |
| Sex | -1.762 | 1.288 | -0.100 | -1.369 | 0.173 |
| Scanner site | 2.226 | 1.164 | 0.135 | 1.913 | 0.058 |
